# Author Correction: Methods and open-source toolkit for analyzing and visualizing challenge results

**DOI:** 10.1038/s41598-021-88636-3

**Published:** 2021-05-06

**Authors:** Manuel Wiesenfarth, Annika Reinke, Bennett A. Landman, Matthias Eisenmann, Laura Aguilera Saiz, M. Jorge Cardoso, Lena Maier-Hein, Annette Kopp-Schneider

**Affiliations:** 1 Division of Biostatistics, German Cancer Research Center (DKFZ), Im Neuenheimer Feld 581, 69120 Heidelberg, Germany; 2 Division of Computer Assisted Medical Interventions (CAMI), German Cancer Research Center (DKFZ), Im Neuenheimer Feld 223, 69120 Heidelberg, Germany; 3 Electrical Engineering, Vanderbilt University, Nashville, TN 37235-1679 USA; 4 School of Biomedical Engineering and Imaging Sciences, King’s College London, London, WC2R 2LS UK

Correction to: *Scientific Reports* 10.1038/s41598-021-82017-6, published online 27 January 2021

In the original version of this Article, Lena Maier-Hein and Annette Kopp-Schneider were omitted as equally contributing authors.

Additionally, Lena Maier-Hein was omitted as a corresponding author. Correspondence and request for materials should also be addressed to l.maier-hein@dkfz-heidelberg.de.

These errors have now been corrected in the PDF and HTML versions of the Article.

